# Repetition and practice. Developing mental training with young violinists: a collaboration

**DOI:** 10.3389/fpsyg.2024.1327763

**Published:** 2024-02-21

**Authors:** Fiona Mary Vilnite, Mara Marnauza

**Affiliations:** Faculty of Education, Psychology and Art, University of Latvia, Riga, Latvia

**Keywords:** mental training, violin teaching, violin learning, practice, collaboration

## Abstract

Mental training has been used successfully by professional musicians and athletes, yet rarely applied in pedagogical processes. As research in neuroscience can now explain how it connects to the processes of learning, its application and adaptation in pedagogy can now be explored. The aim of this mixed methods study was to investigate concepts of repetition and practice with mental training, and discuss adaptations for young violinists, to include attention, awareness, and creative musicality. Three exercises were developed with nine students (average age 8). The first involved creation of imagery, followed by physical practice; the second alternated mental imagery with physical practice; the third involved concurrent use of physical practice and mental imagery. Results of the first exercise indicate heightened awareness of technical skill; self-discovery of bow control, speed and distribution, tone production improvements and an ability to sustain longer notes post-mental training (Z = −2.666, *p* = 0.008 and Z = −2.670, p = 0.008). Observations from the second and third exercises include student experimentation with concepts of musical interpretation, an eagerness to repeat repertoire (≥ 5 times) and increased self-awareness of technical and musical accomplishments. The research suggests that mental training can be adapted for younger learners, that it encourages collaboration in the pedagogical process, and develops student self-awareness of the cognitive and physical processes of violin playing.

## 1 Introduction

Repetition and practice are widely recognised as essential aspects of effective musical instrument training. However, connecting concepts to matters of personal relevance (Willis, 2006), and developing an independent awareness of skill that inspires creative, musical thought (Mawang et al., 2019), can activate a larger network of neuronal connections, thus making learning longer lasting (see Amaral and Fregni, 2021).

The cognitive and mental strategies that assist in addressing these factors, however, are rarely applied deliberately or systematically (see McPherson, 2005) and critics have noted that even modern approaches still incorporate aspects derived from traditional, hierarchical, teacher-centred approaches, including drilling or repetitive learning techniques (Baker, 2014; Baker, 2016). The inadvertent inclusion of these approaches may diminish students’ interest in learning, potentially resulting in accumulative educational trauma identified in a significant number of adult learners (Gray, 2019) and negatively impacting attitudes towards learning and lifelong learning capabilities (Perry, 2006). This is particularly concerning for musicians who need to develop a vast repertoire, maintain a consistently high level, and deal with stresses met with when performing in public. The detrimental effects of these accumulative psychological stresses are evident in cases where musicians resort to medication (Saintilan, 2020), or in instances where musicians experience the exacerbation or triggering of physical injuries, such as focal dystonia (Ioannou et al., 2016). It is therefore necessary to identify aspects that encourage mental and physical well-being and develop pedagogical approaches that encourage student self-awareness of skill in a creative manner from the beginning of learning.

Whilst some current publications note the importance of mental strategies in learning and skill development in music (e.g., Svard, 2023), there is still a lack of literature introducing mental training strategies for young learners and how teachers could include them collaboratively and purposefully in pedagogical processes. Considering its benefits, including cognitive constructs that assist in developing awareness of strategies required to learn new repertoire (see Klöppel, 2010), how could mental training be introduced into learning processes?

For mental training to be incorporated systematically and deliberately into pedagogical processes, it is necessary to identify its foundations, connected neural processes in the brain, and consider how it could include both student personal relevance and collaboration between student and teacher.

The aim of this mixed qualitative-quantitative study, therefore, was to investigate concepts of learning, repetition and practice, its connections to mental training, reveal the necessary adaptations of mental training for young violinists, and devise a set of mental training routines that can be introduced into the primary school violin teaching and learning process that assist in development of students’ violin playing skill. It presents the observations and results of introducing three specifically designed mental training routines within the one-to-one primary school violin teaching and learning process.

### 1.1 Definitions

Mental Training has been defined as the training of mental practice—performing an action in the mind without its physical realisation (Eberspächer, 2007). In the context of music, mental training aims to develop the imagination of both movement and sound (Klöppel, 2010). The process of mental training is based on the manipulation of mental imagery (Budnik-Przybylska et al., 2023), which can take two primary forms:

- Direct imagery: Involves “seeing” in the mind’s eye and employs any single or combination of sense modes: visual, haptic/motoric, auditory, etc. (Thomas, 2016).
- Indirect imagery: Involves metaphoric representations conveying abstract objects or ideas that provide sensory-motor concepts relating to an action/skill (e.g., Short et al., 2001; Pecher et al., 2011; Stergiou et al., 2019), or concepts related to subjects and knowledge (e.g., Saban, 2008; DeSantis et al., 2022).

Mental training can also encompass emotional aspects (Immonen et al., 2012), observation (Mayer and Hermann, 2011), and combine physical movement, such as instrument playing movement, together with mental imagery, including haptic, auditory and other sensory modalities (Connolly and Williamon, 2004; McHugh-Grifa, 2011).

The non-systematic inclusion of mental training in pedagogical processes may be attributed to the lack of uniformity in its labelling (Haddon, 2007). Consequently, even if aspects of it are incorporated, they may not be recognised as such, and therefore, its significance is overlooked. Indeed, one source notes that terms such as “mental training,” “mental rehearsal,” “imagery,” and “visualisation” are used interchangeably without special reference to their implications or meanings (Svard, 2023). However, since this is discussed in relation to terminology involving mental imagery as a method and tool in practicing a musical instrument, it aligns with other sources, affirming that mental training involves training the use and manipulation of mental imagery (see Mayer and Hermann, 2011). Sources have noted that mental imagery plays a key role in assisting predictive thought processes by connecting them to emotions and enabling simulation and exploration of future scenarios. By imagining “placing” oneself in situations and experiencing the resulting emotions, both decision making (Wicken et al., 2021) and motivation for activities (Renner et al., 2019) is facilitated.

**Figure 1:**
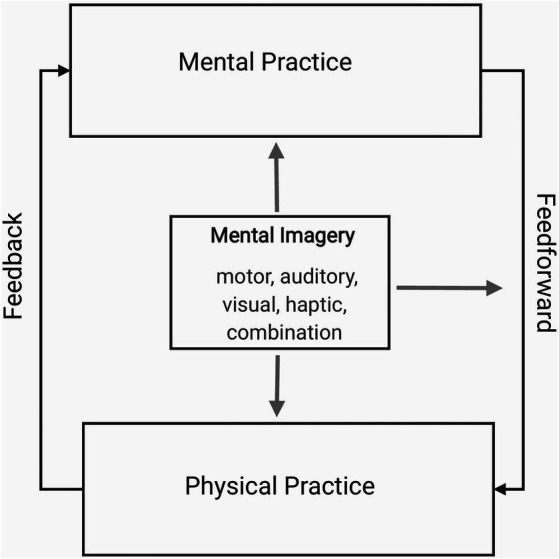
The process of mental training with already-trained practitioners (diagram based on combined concepts of Connolly and Williamon, 2004; Klöppel, 2010; McHugh-Grifa, 2011).

With already-trained practitioners, the process of mental training often involves the alternation of mental and physical aspects: for example, mental practice, which provides feedforward (projection of movement and sound as imagery), followed by physical practice, providing feedback (auditory perception and analysis of the actual sound produced). Figure 1 illustrates this process together with the role of mental imagery: that it is employed during mental practice and feedforward and additionally during physical practice, if physical practice and mental imagery are executed simultaneously, as in playing repertoire in an imaginary concert hall, for instance (see Figure 1).

To understand how to introduce this process to pedagogical approaches and why it would be advantageous, it is necessary to understand how mental training and learning in the brain converge.

### 1.2 Processes of learning in the brain

Learning has been detected through changes in the strength of synaptic connections between neurons in the brain (Owens and Tanner, 2017). The more connections there are, the more efficiently the brain processes and carries out a task—retrieving a memory or executing and repeating an action, for example. Learning influences these connections, making them bigger and more in number; the more learning that is carried out, the more learning is promoted (Willis, 2006). This neuroplasticity—the brain’s ability to change in structure and function, to form and deplete neural connections due to activity and mental experience (Doidge, 2015; Costandi, 2016; Hawkins, 2021)—allows reshaping and reorganising of a network of dendrite-neuron connections (Hawkins, 2021). An important aspect in skill learning, considered to be an aspect of neuroplasticity, is myelination (Xin and Chan, 2020). Produced in response to repeated movement or practice (Ishibashi et al., 2006; Bakiri et al., 2011), myelin is a fatty coating formed on the axons between neurons to allow faster transmission of electrical signals, increasing skill accuracy and smoother, more coordinated movement. Yet, it is not only physical practice that initiates this process. Both mental practice—imagery of movement—and actual physical movement activate many of the same brain areas (Decety, 1996): motor and premotor areas during imagery of motor tasks (Hanakawa, 2016), auditory and prefrontal circuits during auditory imagery, with activation of the right auditory cortex during imagery of instrumental music, correlating to the pitch-processing role in that area (Zatorre and Halpern, 2005). Indeed, training motor imagery—imagery of motor movements—achieves use-dependent plasticity in the brain, similar to that noted after physical movement (Ruffino et al., 2019).

The concept of repetition in skill learning is, therefore, supported in neuroscience: the more a skill is practiced, either mentally or physically, the stronger the neuronal connections for that skill become. Yet, whilst pure repetition would account for some fluency, what about *incorrect* movement? Does a tense movement that perhaps creates an undesirable sound or pitch on the instrument also become reinforced through repetition? This has been discussed in the violin literature (see Deverich, 2013), but as neurophysiologist Aleksandrovich Bernstein noted in the 1930s, the quasi-Pavlovian model of movement learning that emphasises executing only correct movements for effective learning, should be questioned, considering that children learn to ride a bike, for instance, despite many initial mistakes (Ito, 2011). He proposed that actions are goal-driven, with mental representations of actions preceding physical execution; that feedback from the environment derived from physical experience results in error identification and correction, forming an important part in the learning process. Furthermore, Bernstein observed that effectively practicing a motor skill requires focusing on the process of problem-solving, using different techniques and improving upon them with each repetition, rather than simply repeating the same technique (Bernstein, 1967).

Indeed, the cyclic nature of the mental and physical processes of playing a musical instrument has been noted in contemporary neuroscience. In a process that mirrors the one developed consciously in mental training, it starts with reading a musical score or observation, sets in motion a cycle of “feedforward” of sound and movement as the player understands it, in anticipation—before actual production on the instrument—setting a goal for the player. Upon sound production, sensory “feedback” is received, enabling real-time adjustments based on the initial goal or mental model created at the beginning of the process (see Zatorre et al., 2007; Wan and Schlaug, 2010; Keller, 2012; Gruhn, 2015). As the musician continues playing, this whole process repeats and resembles the phenomenon described by musician Barry Green as a “duet” between the music played in reality and the music in the mind (Green, Gallwey, 2015). The musician constantly predicts, then assesses sound and movement, making instant adjustments to achieve the desired outcome—a process that may occur in a split second in reality. Significantly, this intrinsic, spontaneous process occurs, whether or not players are conscious of it. Whilst literature has associated these processes with mental imagery (e.g., Keller, 2012), it is integral to instrument playing and not necessarily employed deliberately as in mental training.

These combined observations have two important implications. Firstly, they highlight that the basis of mental training—mental imagery—is an integral part of mental processes underlying musical instrument learning and cognition. Secondly, that spontaneous mental imagery could be considered in pedagogical approaches and by bringing to attention the occurrence of mental imagery, a sense of metacognition may be developed with the student, enhancing awareness of the cognitive processes associated with learning and how they are related to physical actualisation.

### 1.3 Spontaneous and deliberate mental imagery in the pedagogical process

Spontaneous mental imagery is not limited to that generated when playing a musical instrument, however, its production has been observed also in response to language usage (McCrum, 2016), to questions (see Kosslyn et al., 1995) and metaphors (Benedek et al., 2014). Visual, auditory, or combined visual and auditory imagery can be prompted by questions that involve remembering the attributes of a house or the sound of the washing machine, for example (see Kosslyn et al., 1995). Similarly, sensory imagery can be generated through using metaphors and similes that may compare dynamic contrasts to thunder or a mouse. Indeed, the brain’s processing of figurative language and its connection to mental imagery production has been discussed in the literature: that the use of metaphors involves the activation of sensory and motor areas of the brain responsible for creating mental images corresponding to the metaphorical meaning of the language (Benedek et al., 2014).

Therefore, it is possible that instrument playing technique, structural and interpretive aspects of a musical composition can be illustrated through mental imagery, regardless of whether the imagery is deliberate or spontaneous and that this process could be facilitated by the pedagogue.

According to literature in psychology, there is only a subtle distinction between spontaneous and deliberate mental imagery, with the term “Mental Simulation” being applied to describe imagery that is either deliberate or spontaneous (see Anderson et al., 2019). Literature in cognitive neuroscience noted that both deliberate and non-deliberate mental imagery assists in learning (Pearson et al., 2015) and in psychology, that mental imagery employing metaphors has been associated with creative activities connected to learning (Benedek et al., 2014). Sources in educational neuroscience identified that mental representation, including imagery, metaphors, and similes, activates various brain regions responsible for different attributes of the object, such as size, shape, smell, colour, and emotional association (Wolfe, 2010); the greater the diversity of brain regions or networks of neurons that are activated during learning, the stronger the networks become, thus learning becomes longer-lasting (Amaral and Fregni, 2021).

These important observations establish a link between mental imagery, metaphors, similes, and the concept of neuroplasticity in learning; that mental imagery not only strengthens neuronal connections for particular movements, but also, when used for explaining concepts through similes and metaphors, widens the networks used in learning. These wide-ranging areas of the brain are brought together in the medial temporal lobe (Shimamura, 2010) and the hippocampus (Tan and Amiel, 2022). Recollection of an object or subject being learnt improves as the connections between the brain cells or neurons in a network become stronger through repetition or the re-visiting of the subject content.

So, whilst it can be seen that repetition assists in skill learning, that deliberate and spontaneous mental imagery, along with the use of metaphors and similes, widens and strengthens neural connections, further analysis of the literature suggests the use of imagery not personally relevant to the learner may fail to inspire interest or attract attention, thus making the learning process less efficient. To understand this point, it is necessary to review aspects that mediate attention and awareness in learning.

### 1.4 Personal relevance, attention and awareness

The relationship between attention and cognition, and the ability to focus attention on relevant and ignore irrelevant information, has been discussed in the pedagogical literature (e.g., Robinson, 2012). The identification in psychological and neuroscientific literature that the amygdala, or amygdaloid complex, in the brain is responsible for attaching emotional importance to sensory information has been noted (e.g., Gallagher and Holland, 1994; Sah et al., 2003) and provided a basis for further studies in both pedagogy and neuroscience (e.g., Lu et al., 2020) concerning attention in learning. Indeed, emotions assist in prioritising and bringing to attention information to be learnt and the amygdala attaches emotional significance to memories (Tan and Amiel, 2022).

Interestingly, mental imagery and emotion are closely linked (see Benoit et al., 2019), and so too is the involvement of the amygdala. A network involving the insula, anterior cingulate cortex, amygdala, and ventral basal ganglia activate to encode the significance of a mentally represented experience (Skottnik and Linden, 2019).

Whilst these structures are involved in the production of mental imagery, it is important to note that the amygdala also seems to be involved when negative mental imagery is generated—imagery that envisages negative outcomes in performance (MacNamara, 2018). Negative imagery has been recognised in sports literature as being detrimental to performance (see Morris et al., 2005), and is associated with anxiety and negative performance outcomes in psychology research (Thunnissen et al., 2022). Indeed, anxiety initiates a stress response: muscle tension, activation of the sympathetic nervous system, and the initiation of “fight or flight” reactions, which can impede both retrieval of stored information and knowledge acquisition (see Perry, 2006). Repeated, chronic stress can lead to changes in the hippocampus (Lee et al., 2016)—a brain area associated with learning and memory, which assists in converting short-term memories to long-term memories before being stored elsewhere (Fogwe et al., 2023). Additionally, stress causes degeneration of neuronal dendrites—branch-like connections at the tips of neurons—and a reduction in hippocampal neurogenesis—the formation of new neurons (Lee et al., 2016).

Whilst it is important to keep imagery positive, a moderate amount of short-lasting stress could be beneficial in learning (see Dhabhar, 2018). Positive, but novel situations therefore, such as playing in a concert, where “stress” is of short duration may actually assist in the learning process, contribute to widening the network of neuronal activations, catch attention by creating a novel experience, add emotional significance to the musical repertoire, and create a personally meaningful context for the learning process itself. In fact, novelty, or unexpected stimuli, including an event that occurs out of context, has been identified as being prioritised in both memory and learning processes, since it creates a situation that requires the learner to search for an explanation (Foster and Keane, 2018).

The hippocampus is involved with detection of novelty in the brain—a process that can begin with the prediction of inputs—spatial, temporal or upcoming events, for example, built from knowledge and experience—followed by the actual input, which is then followed by a comparison of predicted versus actual input. A mismatch between predicted and actual inputs creates novelty—the outcome of the comparison of predicted versus actual inputs (Frank and Kafkas, 2021). Novel stimuli have also been identified as activating the amygdala, enhancing sensory processing, perception and, ultimately, cognition (Schomaker and Meeter, 2015).

So, whilst novelty and short-term stress seem to enhance the learning process, prolonged stress is detrimental. Uncertainty, therefore, during preparation leading up to future concerts or exams could create anxiety through imagery of negative situations, thus hindering the learning process. Indeed, mental simulation has been identified as a mediating factor in the relationship between uncertainty and its effects: that whilst positive imagery can occur, humans have a propensity to simulate negative outcomes. However, these simulations are dependent on the type of situations that induce them (Anderson et al., 2019). For instance, uncertain situations, if they inspire optimism, can lead to positive mental simulations, whereas situations that result in pessimism can lead to negative projections of the future (Anderson et al., 2019) which, according to research examining the effects of negative mental imagery, would not create an environment conducive to learning.

These combined observations have significant implications in the teaching and learning process. Firstly, linking subject content to something that has personal relevance or perhaps facilitating the creation of personal relevance helps to attach emotional significance to learning. Secondly, in preparing for concerts and exams, teachers need to assist students to reduce uncertainty, assist in creating positive future projections of events, so that negative mental imagery and therefore also stress may be reduced. Additionally, aspects of novelty could be considered, which could assist in drawing attention to and maintain interest in the subject content. These aspects could be considered as essential to the development of skill in both violin playing and learning.

From analysing the combined literature in neuroscience, pedagogy and mental training, it can be inferred that mental training routines that develop concepts of repetition, practice, and self-awareness of violin playing skill should include:

- A mixture of metaphoric and deliberate mental imagery to assist in the experimentation of concepts and techniques in violin playing.
- Development of interest in repetition and practice, together with awareness of thinking, experimentation, and adjustment of techniques to achieve desired goals.
- Awareness of mental and physical processes connected to feedforward and feedback through the incorporation of mental imagery.
- The use of creative and personally relevant imagery to address attentional processes identified in the developmental cognitive sciences.
- A flexible, adaptive approach, according to the student’s mood and interests.
- Development of clear musical characters and emotional meaning into the music to enhance attentional processes, creating anchors for long-term memory retention.
- Minimisation of negative imagery, avoiding language and behaviour that could trigger negative imagery formation.

## 2 Materials and methods

### 2.1 Design

To address the research objectives, a mixed quantitative-qualitative methodology was employed. Considering the foundations of mental training and its connection to learning processes in the brain, it was hypothesised that mental training can assist in developing concepts of violin playing, repetition and practice if:

- Students maintain interest in a personalised learning process.
- The violin teaching and learning process incorporates specifically adjusted mental training routines.
- Pedagogues employ these routines purposefully in the one-to-one violin teaching and learning process.

The hypothesis, applicable to the quantitative perspective of the research, was accompanied by a research question. This assisted in detailing aspects of the research through pedagogue observation and formed the qualitative aspect of the study:

- What changes are observable in the student’s process of studying during and post the employment of the mental training routines?

To test the hypothesis and to identify the impact of mental training on the development of the concepts of repetition and practice, aspects of technical skill, and musical expression, assessment was conducted before and after mental training. Quantitative data was collected, consisting of two key measurements: (1) the number of repetitions performed by each student, and (2) the duration of sostenuto notes played during both down and up-bow movements. The collected data was analysed using IBM SPSS Statistics software and google sheets, enabling evaluation of the observed changes resulting from mental training. Further observations during the mental training exercises included documentation of the imagery that the students developed and the violin techniques it inspired the students to experiment with.

### 2.2 Participants

Nine violin students from classes 2 to 7 (average age: 8) from a specialist music primary school participated during their scheduled one-to-one lessons. Duration of each lesson: 20 min. The research was carried out in 2017 and adhered to all the ethical guidelines in place at the authors’ establishment at the time—Riga Teacher Training and Educational Management Academy. Written parental consent was therefore sought and obtained. Student participation in the research was voluntary.

The three mental training routines developed for the study were introduced in separate lessons and the routines occurred following the introduction to the lesson, ensuring that the students’ basic needs had been addressed and fulfilled. The mental training routines had not been used with the students prior to this research.

### 2.3 Baseline observations

Observations made prior to the use of the mental training routines revealed several areas where students’ skills could be improved. These included a lack of confidence during concert performances, timid sound production, not playing with a focused sound “in the string,” and uncertainty about the order in which they wished to play their repertoire. Despite discussions and demonstrations in lessons, students had difficulties remembering where to position themselves on the stage in relation to the piano and audience. Students’ playing lacked variation in musical character and dynamics, use of sound points (where on the string to place the bow to create different sound qualities), awareness of bow distribution (in which sections of the bow to play), bow speed and right arm weight.

Indications of improvement of skill after mental training could therefore include increased awareness of bow distribution and right arm weight, musical colour and character, and increased confidence during playing. To assist in determining an aspect of this quantitatively, students were asked to play a long note throughout the entire bow on both up-and down-bows and to sustain it for as long as possible. The number of beats a student could play with a metronome beat of 80 BPM was notated (see Table 1).

**Table 1:** The length of students’ sostenuto on down and up bows at 80 BPM before mental training.

Student	Down bow count (80 BPM)	Up bow count (80 BPM)
**1**	12	13
**2**	9	9
**3**	11	14
**4**	8	7
**5**	4	4
**6**	8	7
**7**	3	7
**8**	40	46
**9**	10	9

A common issue noted during this task was that students were tensing their upper right arms, particularly when playing near the heel of the bow, resulting in students attempting to lift the entire right arm to produce a lighter sound at the heel, rather than allowing the elbow of the right arm to drop naturally. This would have enabled them to lighten the hand, producing a less “pressed” sound effortlessly.

Baseline observations also revealed that a thorough review of the basic bow hold was necessary. The students needed to focus on bending their thumbs and placing a “curved” little finger on top of the bow. Bending the thumb would facilitate a relaxed position for the hand whilst playing near the heel as well as during bow changes, enabling arm weight to enter the bow when playing at the tip.

The number of times a student was willing to repeat their repertoire, or phrases within their repertoire, during the baseline observations was also recorded (see Table 2).

**Table 2:** Number of repetitions of repertoire before mental training.

Student	1	2	3	4	5	6	7	8	9
No. of repetitions	3	2	3	1	1	2	2	1	2

Students were asked to repeat repertoire, or areas of it, for improvement of tone production, intonation, rhythm, dynamic contrasts, for example. Issues observed during the repetitions included a lack of attention and a tendency to become easily distracted, especially with student 4, who was distracted by an interest in the classroom piano, and student 5, who began talking about unrelated issues. These students evidently did not yet wholly grasp the purpose of repetition, finding difficulties identifying areas requiring improvement, even when the teacher provided explanations. A lack of attention to detail noted amongst all students suggested a need to refine concepts of an ideal sound, plus perhaps the need to lose any possible fear of making mistakes.

### 2.4 Mental training routines

Based on the analysis of the literature in neuroscience, psychology and pedagogy, three mental training routines were developed. Routines one and two were based on literature connected to attention, the use of personally relevant imagery and development of self-awareness of violin skill. The third routine, based on the traditional mental training exercise of mentally rehearsing the concert process, paid attention to building positive performance associations and scenarios, shown in the literature to be effective at reducing uncertainty whilst preparing for concert situations.

#### 2.4.1 First routine: “Elephant, Elephant”

Materials needed: A metronome at 80 BPM on silent intended for use by the teacher. Paper and pen/pencil.

The purpose of this routine is twofold: firstly, to develop awareness of violin playing skill, particularly bow direction, bow speed, right arm weight and awareness of different regions of the bow—aspects connected to tone production that can be challenging to approach and maintain student interest in the early stages of learning the violin. Secondly, the routine aims to introduce the concepts of repetition and experimentation and to develop the understanding that skill can be improved with each repetition.

**Figure 2:**
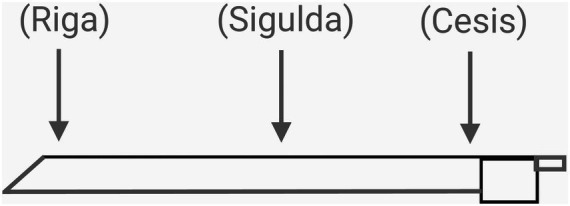
The violin bow split into three areas.

This routine begins with an introductory phase, where the teacher and student examine the bow hold. Using imagery and similes for describing the position of the fingers and hand, such as “a bird perched delicately on a branch” for the rounded position of the right-hand finger on top of the bow and a “dolphin that is swimming upwards” for the position of the thumb under the bow, the student and teacher build the correct bow hold. The teacher also encourages the student to develop imagery themselves. The use of imagery is then extended to conceptually separate the bow into three areas (see Figure 2). This is achieved in steps 1 to 3 below.

1. Building imagery for the heel of the bow. Since the bow is held by the heel of the bow, the student is asked to imagine that their hometown (Cēsis) is by the heel of the bow. Because the bow will be moving in order to produce a sound, the teacher asks the student to imagine a train (or car) travelling from their hometown (Cēsis) and shows the area of the bow by the heel.
2. Building imagery for the tip of the bow. The teacher points to the tip of the bow and asks the student to imagine where the train could travel to by the time it reaches the tip of the bow (e.g., the capital city Rīga).
3. Identifying the midpoint of the bow. The teacher asks the student which town is halfway between their hometown (Cēsis) and the capital city (e.g., Sigulda).
4. Imagery to assist in developing right arm balance and weight. Imagery of elephants travelling on the train is offered by the teacher to give the feeling of weight in the hand and arm as the bow travels throughout the length of the bow and to assist in producing a stable sound “in the string.” The teacher says, “Can you imagine how many elephants could get on the train from Cēsis to Rīga? Let us count them!” The teacher demonstrates playing a down bow, whilst counting “Elephant, elephant…” The teacher says, “I think I had about 4 elephants. How many do you have?”
5. The student plays a “down bow”—a sound starting in Cēsis (by the heel of the bow) and travels with the bow (or “train”) towards Rīga (the tip of the bow). The teacher counts aloud, “Elephant, Elephant, Elephant” whilst the student plays. One “elephant” is equal to one beat at 80 BPM. (The teacher observes the metronome in silent mode to count the beats accurately.)
6. The teacher and pupil discuss the number of elephants counted.
7. The student now plays an “up bow” from Riga to Cesis (from the tip to the heel), whilst the number of elephants is again counted.
8. The number of elephants is compared for down bows and up bows, to ascertain whether one direction included more elephants (or played for a longer duration) than the other.
9. The student is now asked to “travel” on a down bow from Cēsis (heel) to Sigulda (middle) and from Sigulda (middle) to Riga (tip).
10. Analysis of results by the student and teacher and repetition of steps 5 to 10 of the routine.

The number of elephants or beats are notated in a table, for easier subsequent analysis by the student (see Table 3).

**Table 3:** Bow distribution table for routine one.

Bow distributions	No. of elephants / beats (♩ = 80)	
	1st time	2nd time
Cesis—Riga (down-bow)		
Riga—Cesis (up-bow)		
Cesis—Sigulda (down-bow from heel to middle of bow)		
Sigulda—Riga (down-bow from middle of bow to tip)		
Riga—Sigulda (up-bow from tip to middle)		
Sigulda—Cesis (up-bow from middle to heel)		

#### 2.4.2 Second routine: “Emojis”

Materials needed: 5 pieces of sticky note paper, pen/pencil.

The purpose of this routine: to develop musical characters and awareness of musical interpretation; to assist with creating interest in concepts of practice and the “feedforward” of sound and technique.

**Figure 3:**
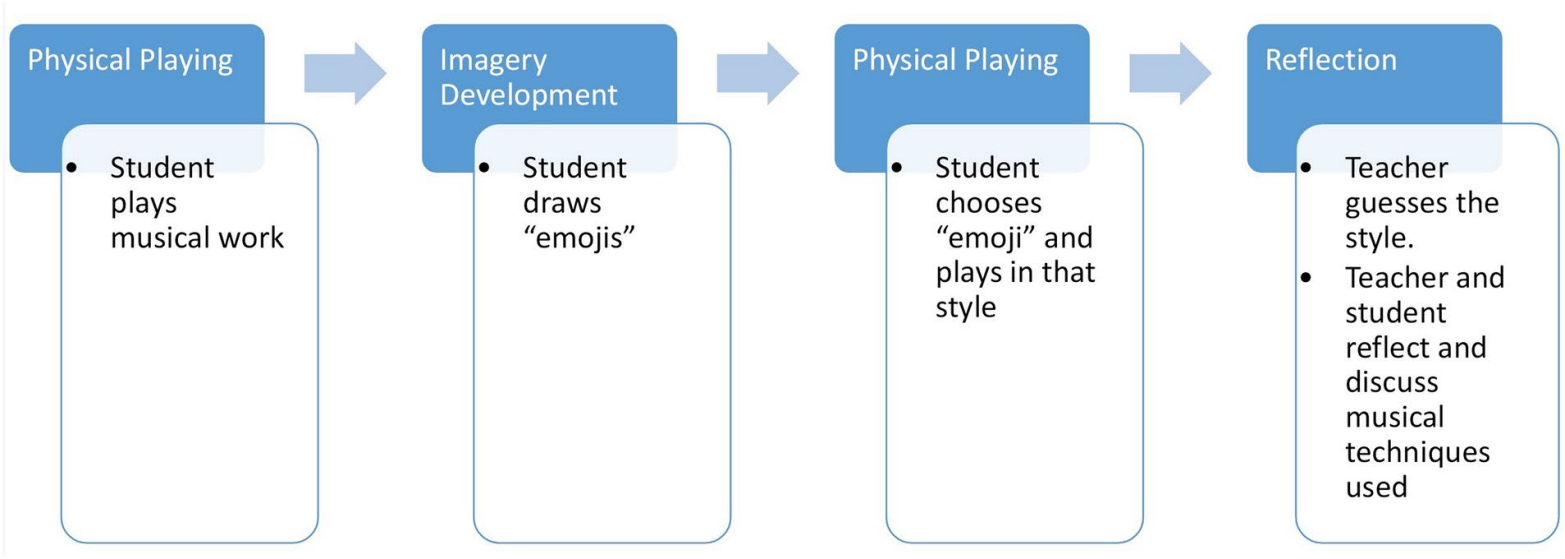
The process of routine 2 (*Emojis*).

1. The student plays a musical work, or a section of a piece they are currently learning.
2. The student draws five different “emojis” showing different emotions on five separate pieces of sticky note paper.
3. Without revealing the order to the teacher, the student arranges their drawn “emojis” on the music stand.
4. The student plays again, and the teacher attempts to guess the emotion being portrayed by the student.
5. Repeat step four 5 times (or as many times as there are emojis).
6. Teacher and student discuss the different musical effects and violin techniques explored (see Figure 3).

#### 2.4.3 Third routine: rehearsing the concert process

The purpose of this routine: to develop concepts of mental rehearsal, memorisation, build confidence leading up to performance, and draw attention to the learning process. To encourage positive concert and playing scenarios, the teacher uses positive imagery of the concert situation. The development of imagery for the concert process involves a combination of action and imagery.

1. Student and teacher exit the classroom and close the door. The teacher guides the student through the time before entering the stage, describing how to safely hold the violin in resting position, preferably under the right arm, so that one arm is free to hold on to any bannisters, if there are steps, or to open or close doors, etc.
2. The teacher describes an imaginary scene of the stage and the circumstances that might precede the student’s performance. For instance, the student may need to wait for the previous performance to end before playing/entering the stage, so the teacher can ask the student to listen to ascertain whether there is anyone still playing.
3. Teacher and student enter the classroom as if entering the stage. The teacher asks the student to imagine the audience who are now clapping (the teacher may also clap to imitate the audience). In order to remind the student to bow to the audience, the teacher asks, “What do we have to do when the audience is clapping?”
4. The student prepares to play. Still imagining the direction of the imaginary audience, the student can be prompted to stand with the f-holes of the violin facing the audience, in a location where the student can also communicate with any accompanist.
5. The student plays the work(s) in the same order as will be required in the concert/exam. If there are several works, the student and teacher discuss what will happen in the gaps between the pieces: where the violin will rest and whether or not to bow to the audience.
6. After the musical works are played, the student is asked about post-performance actions on stage. The student can be reminded to smile (or imagine smiling), to bow before exiting the imaginary stage, whilst envisioning applause from the audience. Teacher and pupil again exit the classroom as if exiting the stage.
7. Analysis of the various aspects of this process follows. Using positive language and after congratulating the student for completing the process, the teacher asks how the student feels, whether they feel they have achieved their goals; whether anything went as expected, or if anything was surprising.

This entire routine can be repeated until the student becomes comfortable with creating imagery of the concert situation. It is envisaged that the exercise will become a reference point for subsequent mental rehearsals and can be repeated in subsequent lessons. In a lesson plan this routine could be placed at the end of the lesson to review achievements or near the beginning of the lesson for attentional aspects and adapting to the lesson environment.

## 3 Results

### 3.1 Teacher observations during the mental training routines

Routine no.1: “Elephant, Elephant.” Students were happy to “invent” three separate areas on the bow. The students also became interested in the silent electronic metronome that the teacher was holding, which the teacher then showed the student. This routine became most effective when taking a break between repertoire, or when it seemed like a student’s concentration was wandering and provided a method of focussing. The students were keen to find out how many elephants had been counted on each bow. This also seemed to inspire the concept of repetition, since students were keen to experiment to see whether they could increase the count on subsequent repetitions of the routine without any prompting from the teacher.

Routine No.2: “Emojis.” This seemed to calm the students, reducing self-doubt that occasionally affected fluency during playing. Two students were unfamiliar with the term “emojis,” prompting an explanation that they represented “faces” and “expressions.” Some students asked whether they could also draw animals.

The younger students were content with playing a single line of music and then asking the teacher to guess their characters. Some students asked why they needed the emojis; the teacher explained that they help to play the music in different ways or characters. The teacher then took the violin to demonstrate techniques and sound qualities that could portray a happy or sad mood, explaining that that was only one way to express those emotions; that they could experiment with their own sounds and emotions. Interestingly, all students were keen that the teacher should also draw emojis. No emphasis was placed on the concept of repetition or practice by the pedagogue, since it was hoped that this would be a positive consequence of the experimentation process.

Routine No.3: “Rehearsing the concert process.” This seemed effective in addressing attentional aspects. The novelty and element of surprise at the beginning when exiting the classroom seemed to increase concentration after re-entering the classroom.

The students were happy to imagine the concert hall or exam situation and many of them posed questions about where they should stand and which piece they should perform first. Those who did not ask questions or who were not aware of issues were prompted by the teacher, asking questions, such as, “where do you stand?” and “remember that you need to do what (smile, bow to the audience, etc.) before playing?”

### 3.2 A comparison of the results before and after mental training

#### 3.2.1 Routine 1: “Elephant, Elephant”

**Figure 4:**
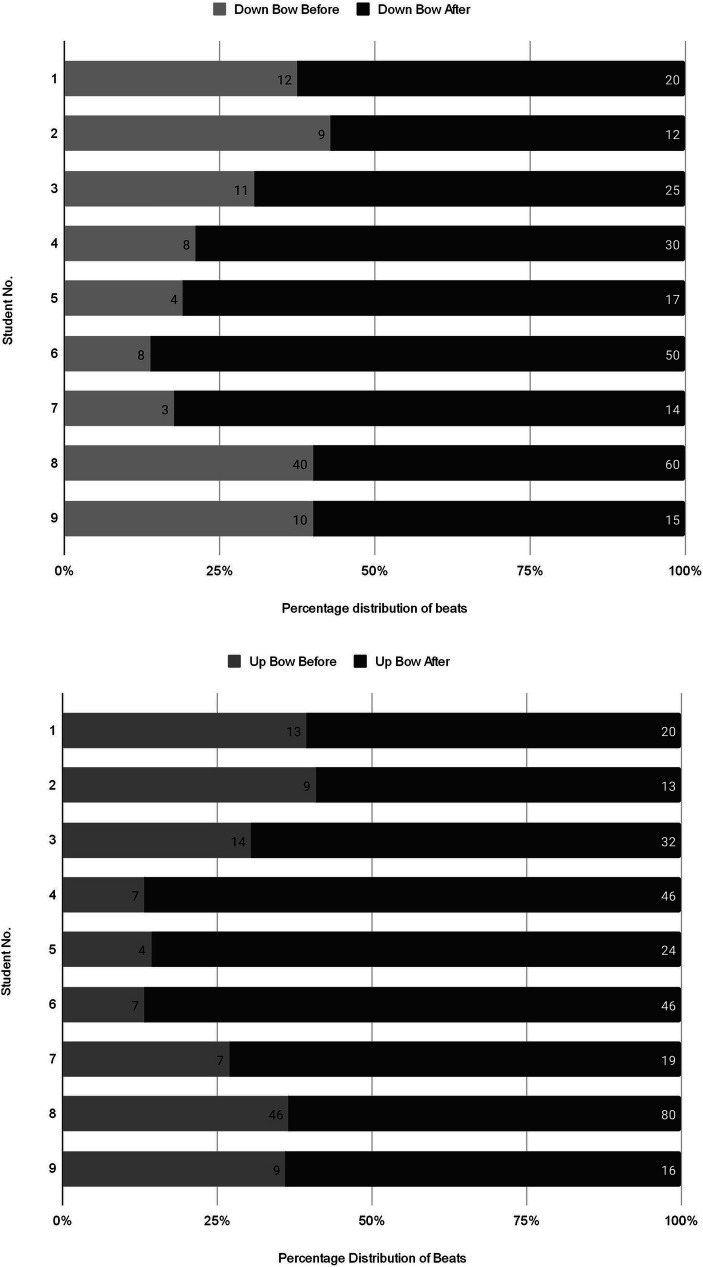
Comparison of the number of beats at 80 BPM for down-bows and up-bows before and after mental training routine 1 “Elephant, Elephant.”

Whilst there were visible improvements in right hand posture and arm weight, comparing the number of beats that a note could be played sostenuto on one bow before and after the mental training routine confirmed that students were able to sustain a longer note post-versus pre-the mental training (see Figure 4). Figure 4 presents a visual depiction of the percentage distribution of beats for the Down Bow technique. That is, it shows the percentages of the beats relative to the total number of beats for each student before and after mental training for each individual student. The bars on the graph represent the count or number of beats achieved for each student.

The most significant changes post routine were noted with students 3, 4, 5, 6, and 7, but all students had increased their bow control with improvements in both speed and weight. Significantly, students had managed to do this using imagery and were happy to experiment themselves and repeat the exercise, without needing lengthy technical explanations. Student 8, who had observed the routine with a different student whilst waiting for their lesson, sustained very long notes even on their first try, suggesting that observation alone had an effect, corroborating other research noting the positive effect of observation in learning and mental training.

All of the students enjoyed analysing the completed bow distribution table (see Table 3) created during the routine. Interestingly, when examining the table, the students identified areas of the bow where their counts were shorter and were keen to increase counts in those specific regions. This was observed universally, even amongst the younger students, indicating that the students had understood the concept of bow distribution.

The longer notes achieved by the students post the mental training routine not only demonstrate an improvement in violin playing skill, but also reflect the results of their experimentation with mental imagery during the mental training routine. Importantly, the repetition of the routine was initiated by the student without any prompting from the teacher, suggesting that the routine kept the students’ attention, inspired independent experimentation, thereby developing concepts related to improvement of skill through practice and repetition.

A Wilcoxon signed-rank test conducted on all the students’ bow counts revealed a value of p of 0.008 (Z = −2.666) for down bows and a value of p of 0.008 (Z = −2.670) for up bows, showing statistically significant changes overall post the mental training routine (see Table 4). This statistical analysis further demonstrates the effect of the mental training routine in enhancing bow control and concepts of repetition and practice across all the students.

**Table 4:** Down and up bows before and after mental training routine no. 1.

Student	Down bow count (80 BPM)		Up bow count (80 BPM)	
	Before	After	Before	After
**1**	12	20	13	20
**2**	9	12	9	13
**3**	11	25	14	32
**4**	8	30	7	46
**5**	4	17	4	24
**6**	8	50	7	46
**7**	3	14	7	19
**8**	40	60	46	80
**9**	10	15	9	16
**Wilcoxon signed-rank test**	*Z* = −2.666 *p* = 0.008		*Z* = −2.670 *p* = 0.008	

Other observations noted after the mental training routine included the development of keywords for quick reference to different techniques: In subsequent lessons, for instance, the terms “Cesis,” “Sigulda,” and “Riga” were adopted as keywords to denote the division of the bow into sections and describe specific areas of the bow to play, being utilised by both teacher and student. Students began to ask questions about where in the bow passages could be played and were ready and able to experiment deliberately with playing in different parts of the bow. Students were also keen to repeat the mental training exercise in subsequent lessons, where further subdivisions of the bow were also discussed.

#### 3.2.2 Routine 2: “Emojis”

Since the purpose of this routine was to develop musical characters and awareness of musical interpretation, as well as concepts of practice and “feedforward,” the students’ drawings were collated, catalogued and described alongside the musical and technical techniques the drawings inspired. The drawings became a quasi-feedforward of the violin techniques utilised by students, assisting them in formulating questions of how their images could be expressed in sound. In effect, the drawings gave the students the opportunity to problem solve—an important aspect of practice noted in the literature.

The drawings created by the students in this routine were translated into keywords (e.g., happy, sad, dog, cat). The keywords were notated for each student and analysed together with the violin techniques employed (see Table 5). Out of the 45 emojis drawn by the students, 31 were unique, indicating the individuality of ideas amongst the students. Only 8 emojis were repeated: Happy: 3 (occurrences), Sleepy: 3, Angry: 3, Dog: 2, Neutral: 2, Shock: 2, Scared: 2, Sad: 3.

**Table 5:** Imagery and violin techniques employed by each student.

Student	Imagery	Techniques	Student	Imagery	Techniques
**1**	Cat	Legato, longer bows	**2**	Happy	Smooth, even bow strokes (*detaché*), bowing midway between bridge and fingerboard
	Dog	Short loud bow strokes (*martelé*), bowing midway between bridge and fingerboard, *forte*		Neutral	Bowing towards fingerboard (*sul tasto*), *piano*
	Happy	Stable sound, bowing midway between bridge and fingerboard		Angry	Short, loud *martelé* bow strokes, middle of bow
	Hare	Heavy, slow, short *spiccato* bow strokes, middle of bow		Sleepy	Slow tempo, bowing over fingerboard (*sul tasto*)
	Heart	Smooth bow changes, slurred notes		Shock	Short strokes, all up-bows
**3**	Sleepy	Slow tempo, bowing over the fingerboard (*sul tasto*), *piano*	**4**	Emoji with sunglasses	Bowing on the bridge (*sul ponticello*)
	Shock	Bowing over the bridge (*sul ponticello*)		Happy	Musical work transposed a 5th down
	Scared	Tremolo, *sul ponticello*		Angry	Short, loud *martelé* strokes, middle of bow
	Happy	Stable, focussed sound		Sad	Slow tempo, played near the point of bow, *piano*
	Sad	Played a 5th down, slower		Asleep	Bowing over fingerboard (*sul tasto*)
**5**	Light	Tremolo bowing, towards the bridge	**6**	Thinking	Slow bows, towards the fingerboard
	Scared	Slow, short bow strokes, slow tempo		Tongue out	Upwards glissandi
	Dark	Notes played on lower strings		Emoji with dollar signs in eyes	Stable sound, bowing midpoint between bridge and fingerboard
	Cold	Tremolo on the bridge (*sul ponticello*)		Pizza	Pizzicato
	Hot	Heavy bow strokes (*detaché*)		Ice Cream	Stable, smooth sound, bowing midpoint between bridge and fingerboard
**7**	Thinking with glasses	Accurate bow distribution and intonation	**8**	Ghost	Fast, floating bow, over fingerboard (*sul tasto*)
	Long nose	Irregular pulse		Skull	Playing above string with the bow
	Night time	Smooth sound, all notes in slurs		Pixelated Minecraft Character	Short, heavy bow strokes (*martelé*)
	Sleepy	Medium fast bow strokes (*sul tasto*)		Neutral Emotion	Gentle sound over the fingerboard (*sul tasto*)
	Fire	Fast, long, bow strokes, towards the fingerboard		Emoji with hearts in eyes	Glissandi upwards between each note
**9**	Surprise	Tremolo			
	Angry	Short bow strokes, all downbows			
	Crying	Glissandi downwards after each note			
	Emoji with sun hat	Slow vibrato with left hand			
	Dog	Fast, long bow strokes			

**Figure 5:**
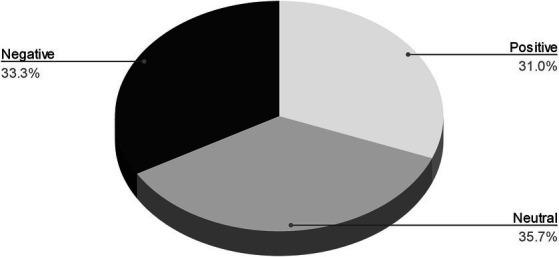
Frequency of positive, neutral and negative sentiments.

Students used varying emotions in their imagery: student 2, for instance, created four images that represented opposite emotions and one neutral emotion: “happy” and “angry”; “sleepy” and “shock” and a “neutral” emoji with an expressionless mouth. These concepts inspired experimentation with contrasting dynamics and violin techniques. Interestingly, when categorising the emojis according to sentiment (positive, negative and neutral), all three categories were fairly evenly represented throughout the 9 students (see Figure 5).

A quasi-co-occurrence matrix was used to detect the presence or absence of a sentiment for each student (see Table 6). “Yes” indicates the presence of a sentiment for a particular student, whilst empty cells indicate the absence of that sentiment.

**Table 6:** Co-occurrence matrix for the sentiments associated with the drawings created by the student.

Student	Positive	Neutral	Negative
1	Yes		
2	Yes	Yes	Yes
3	Yes		Yes
4	Yes		Yes
5		Yes	Yes
6	Yes	Yes	
7		Yes	
8	Yes	Yes	
9	Yes		Yes

Based on the co-occurrence matrix, the following observations can be made, that:

- “Positive” sentiment is present in students 1, 2, 3, 4, 6, 8 and 9.
- “Neutral” sentiment is present in students 2, 5, 6, 7 and 8.
- “Negative” sentiment is present in students 2, 3, 4, 5, and 9.

Not all sentiments are present for every student; there were two students that had only one category of sentiment: happy (student 1) and neutral (student 7), there were no students that had only negative imagery. However, even the students that created imageries categorised as only having one sentiment (such as students 1, 5 and 7), the imagery was contrasting in different ways—such as the difference of sound created by a cat versus a dog (student 1) and light versus dark (student 5) and the concepts of sleeping versus fire (student 7).

**Figure 6:**
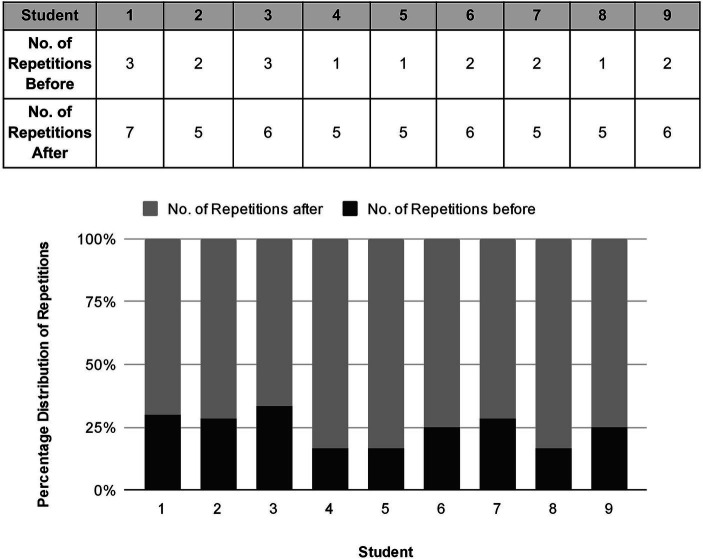
Number of repetitions before and after imagery creation.

Overall, this mental training routine encouraged experimentation with differing musical effects and violin techniques. The “discovery” of the differing sound points (points in the area between the bridge and fingerboard where the bow transverses the strings) provided a creative introduction to technique that is usually considered in the later stages of violin learning, plus experimentation with concepts, such as glissandi and tremolo, not found routinely in the repertoire at this stage. This experimentation assisted the students in becoming more comfortable with their instruments technically and musically. The routine also provided a purpose for repeating and practicing their repertoire and/or work on sections. This occurred at least five times using the emojis created by the students (see Figure 6).

Interestingly, the students also invited the teacher to participate by asking them to draw and play in a similar process, after which some students asked to play their repertoire again, this time in the style of the teacher’s emojis, and incorporated additional techniques they had observed in the teacher’s performance. Eight out of nine students also wanted the teacher to play some repertoire with their emojis, so that they could also guess the emoji in which the teacher was playing.

The students took the emojis they created home and were told that they can continue to practice in this way and draw different emojis. All of the students brought the previously drawn emojis with them to their next lesson, and three of the youngest students had created artwork at home on larger pieces of paper (depicting a violin, a cat and a rabbit and a unicorn). This artwork was then incorporated into their lessons to inspire further musical interpretations.

#### 3.2.3 Routine 3: rehearsing the concert process

This routine proved valuable in focusing attention at the beginning of the lesson, after the initial introduction, to gain student interest in the subject, or towards the end of the lesson, to review achievements in the lesson. Students were intrigued when the teacher suggested leaving the classroom with the violin for “pre-visualising” the concert process. This also seemed to enhance attentional processes. By changing the “pace” of the lesson, students that were otherwise prone to distractions were able to continue to focus better on their repertoire.

Awareness also improved during this routine: students began to detect their own intonational and rhythmical mistakes. As a result, students repeated their repertoire with personally created goals—such as entering the stage and playing the repertoire without mistakes or hesitations. It is important to note that before this they had not consistently reacted upon these aspects in their playing. Perhaps the small amount of stress, as identified in the literature, created when imagining the concert situation helped to clarify the goal of learning the repertoire and facilitated perception from a different angle—perhaps as if they were listening to a performance themselves.

Additionally, this routine seemed to inspire students to play from memory. They began checking their sheet music before repeating the routine (without playing) in order to perform more accurately. Some of them quietly sang their identified “problem” area(s), or checked the correct fingerings, etc. In doing so, they achieved something closer to mental training performed by already-trained practitioners. Students also became more confident in performing in subsequent concerts and exams post this routine.

## 4 Discussion

The routines were designed to prepare the foundation of mental training by attempting to create a conscious link between mental and physical processes connected to playing and learning the violin. Though perhaps not unexpected, it was interesting to note how attentional aspects improved during the routines and how the tendency decreased for students to “drift off,” with their imagination now being directed towards aspects connected to the subject at hand.

Whilst this research did not seek to discover the ideal placement of each routine in the lesson, it would seem that the placement of the routines needs to be judged by the pedagogue in response to the individual needs and mood of the student.

Further research could explore the choice of words that the teacher uses during the routines. Starting sentences with words and questions, like “Remember to…” and “What do you need to do?” (rather than, “do not forget to……” and “why did you not …?”) seemed to assist students to become more positively involved in the process of playing and learning.

Additionally, it was interesting to assess how the number of repetitions became irrelevant, when the students had a goal to attain. Any fear they might have had of making mistakes was also dispelled.

## 5 Conclusion

The results of the mental training routines confirm the hypothesis of the research, that mental training can assist in introducing concepts of violin playing, repetition and practice in a creative, personally relevant and collaborative manner to one-to-one violin teaching and learning processes of young violinists.

Its expansion into the primary school pedagogical process was indeed supported by identifying the foundations and neural processes connected to both learning and mental training.

The research highlighted the significance/interchangeability of both deliberate and spontaneous imagery; the importance of keeping approaches positive and optimistic, and creating a context for studying that supports problem solving and experimentation and that this can be facilitated by the pedagogue.

Results indicate that throughout the routines students were becoming more aware of their own playing skill and thinking processes. They were able to repeat and discuss the way they achieved a certain sound for a particular emotion or effect, suggesting that imagery helped provide a purpose for repetition, which in turn paved the way for purposeful experimentation.

The routines seemed to increase awareness of mistakes, but at the same time facilitated the understanding that mistakes provide information and feedback on the way to achieving a personally relevant, and personally set, goal.

Throughout subsequent lessons and as students began to ask more questions—where in the bow sections of the repertoire could be played, how to create a fluffy or misty sound, etc.—it was possible to conclude that they were becoming more interested in the learning process and that a more collaborative teaching and learning process was achieved. Additionally, the fact that students were keen to also involve the teacher in the routines—such as drawing emojis—suggests that students were comfortable enough to initiate discussion and perhaps viewed the teacher as a companion or mentor within the learning process. Students also began experimenting more independently with different sound points (points in the area between the bridge and fingerboard) and bow speeds, indicating that they were also beginning to construct their own mental models of sound and characters, which is at the basis of independent practice and creative musical interpretation.

Overall, these findings not only expand and corroborate earlier mental training literature, but also provide a means of applying insights from neuroscience and psychology to primary school pedagogical approaches, which may also hold significance in subjects beyond music learning and violin playing.

## Data availability statement

The original contributions presented in the study are included in the article/supplementary material, further inquiries can be directed to the corresponding author.

## Ethics statement

Ethical approval was not required for the study involving humans in accordance with the local legislation and institutional requirements. Written informed consent for participation in this study was provided by the participants' legal guardians/next of kin.

## Author contributions

FV: Conceptualization, Data curation, Formal analysis, Investigation, Methodology, Writing – original draft, Writing – review & editing. MM: Conceptualization, Methodology, Project administration, Supervision, Writing – review & editing.
